# Coronary artery bypass grafting versus stent implantation in patients with chronic coronary syndrome and left main disease: insights from a register throughout Germany

**DOI:** 10.1007/s00392-021-01931-x

**Published:** 2021-08-28

**Authors:** Peter Stachon, Klaus Kaier, Philip Hehn, Alexander Peikert, Dennis Wolf, Vera Oettinger, Dawid Staudacher, Daniel Duerschmied, Andreas Zirlik, Manfred Zehender, Christoph Bode, Constantin von zur Mühlen

**Affiliations:** 1 Department of Cardiology and Angiology I, University Heart Center Freiburg-Bad Krozingen, Faculty of Medicine, University of Freiburg, Freiburg, Germany; 2 Center of Big Data Analysis in Cardiology (CeBAC), Department of Cardiology and Angiology I, University Heart Center Freiburg-Bad Krozingen, Faculty of Medicine, University of Freiburg, Freiburg, Germany; 3 Institute of Medical Biometry and Statistics, Faculty of Medicine and Medical Center, University of Freiburg, Freiburg, Germany; 4 Department of Cardiology, University Hospital Graz, Graz, Austria

**Keywords:** Left main coronary artery disease, Stent, Coronary artery bypass grafting, mortality

## Abstract

**Background:**

Recent randomized controlled trials have sparked debate about the optimal treatment of patients suffering from left main coronary artery disease. The present study analyzes outcomes of left main stenting versus coronary bypass grafting (CABG) in a nationwide registry in patients with chronic coronary syndrome (CCS).

**Methods:**

All cases suffering from CCS and left main coronary artery disease treated either with CABG or stent, were identified within the database of the German bureau of statistics. Logistic or linear regression models were used with 20 baseline patient characteristics as potential confounders to compare both regimens.

**Results:**

In 2018, 1318 cases with left main stenosis were treated with CABG and 8,920 with stent. Patients assigned for stenting were older (72.58 ± 9.87 vs. 68.63 ± 9.40, *p* < 0.001) and at higher operative risk, as assessed by logistic EuroSCORE (8.77 ± 8.45 vs. 4.85 ± 4.65, *p* < 0.001). After risk adjustment, no marked differences in outcomes were found for in-hospital mortality and stroke (risk adjusted odds ratio (aOR) for stent instead of CABG: aOR mortality: 1.08 [95% CI 0.66; 1.78], *p* = 0.748; aOR stroke: 0.59 [0.27; 1.32], *p* = 0.199). Stent implantation was associated with a reduced risk of relevant bleeding (aOR 0.38 [0.24; 0.61], *p* < 0.001), reduced prolonged ventilation time (aOR 0.54 [0.37 0.79], *p* = 0.002), and postoperative delirium (aOR 0.16 [0.11; 0.22], *p* < 0.001). Furthermore, stent implantation was associated with shorter hospital stay (− 6.78 days [− 5.86; − 7.71], *p* < 0.001) and lower costs (− €10,035 [− €11,500; − €8570], *p* < 0.001).

**Conclusion:**

Left main stenting is a safe and effective treatment option for CCS-patients suffering from left main coronary artery disease at reasonable economic cost.

**Graphic abstract:**

**Figure:**
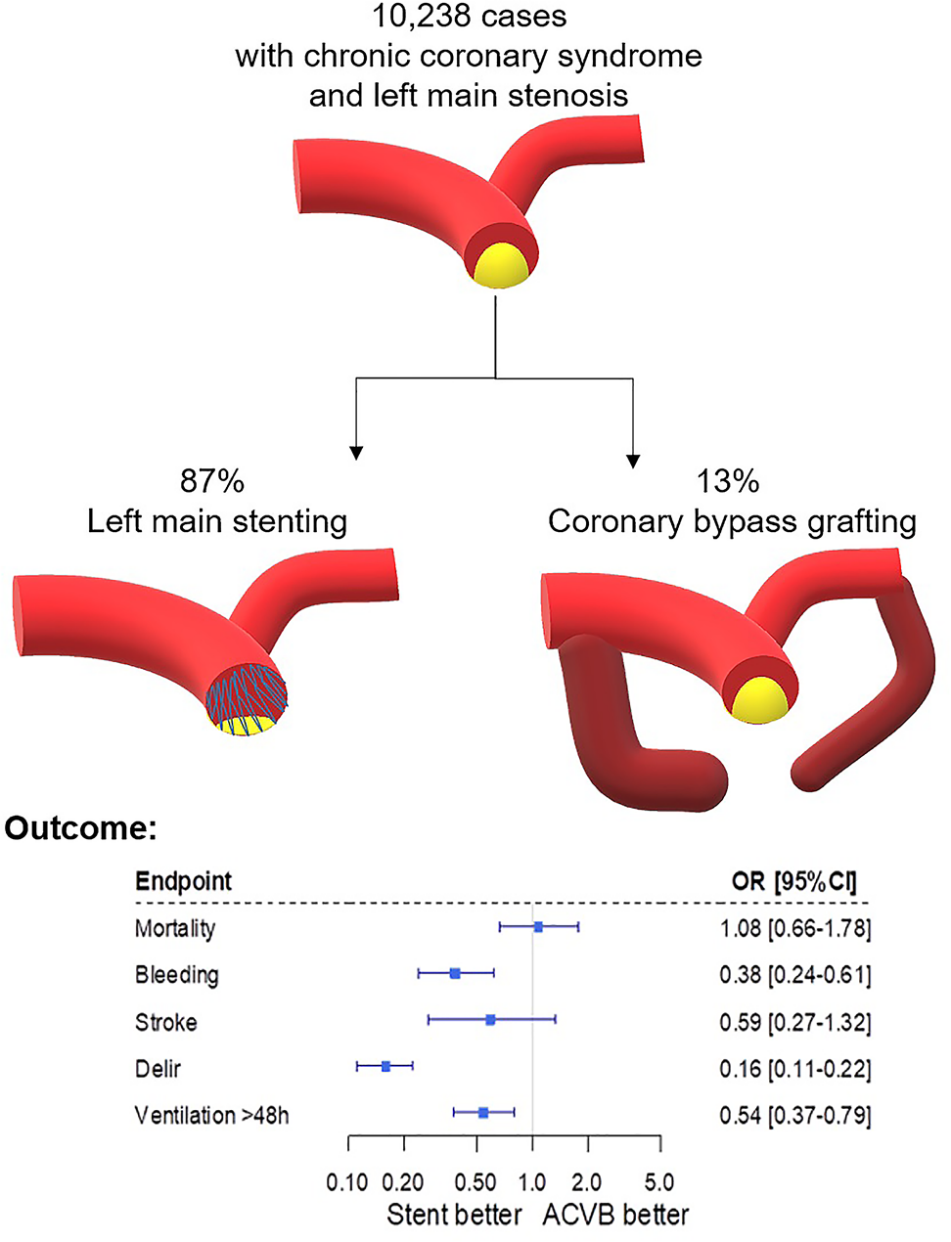
Coronary artery bypass grafting versus stent implantation in patients with chronic coronary syndrome and left main disease: insights from a register throughout Germany. All cases with chronic coronary syndrome and left main stenosis treated in 2018 in Germany either with left main stenting or coronary bypass grafting were extracted from a nation-wide database. In-hospital outcomes were compared after logistic regression analysis.

## Introduction

About 5% of patients with chronic coronary syndrome (CCS) and 7% of patients with acute myocardial infarction suffer from left main disease [9]. The left main coronary artery accounts for about 75% of the blood supply of the left ventricle [10]. Consequently, 3-year mortality of left main stenosis is nearly 50% in patients receiving medical treatment only [9]. Studies from the 80 s and 90 s showed improved survival in patients undergoing surgical revascularization by CABG, with a risk reduction of about 70% [1, 18, 27, 28]. Thus CABG became the standard of care for patients with left main stenosis. However, there was still significant CABG related morbidity and mortality [8]. In 1986 the first coronary stent was implanted. This was a breakthrough for interventional cardiology, since it overcame the weakness of pure balloon angioplasty in particular in left main stenosis [7, 21]. Further innovations and increasing experience in the field of coronary interventions led to the consideration in the last decades whether the less invasive left main stenting may be an alternative to CABG.

Five randomized controlled trials have been conducted with a long-term follow-up ranging from 5 to 10 years [2, 5, 12, 14, 15, 17, 23, 24, 26]. A meta-analysis summarizing those studies found no differences in all-cause mortality, cardiac death, stroke, or myocardial infarction between CABG and percutaneous coronary intervention (PCI) [2]. However, the risk of unplanned revascularization is consistently higher in patients treated with PCI in all studies.

Consequently, the guidelines have implemented those results into their recommendations: PCI and CABG are rated as equivalent by the European Society of Cardiology, with an IA recommendation in patients with morphological low risk stenosis (SYNTAX 0–22). With more morphologically complex stenosis, the recommendations favor CABG [16].

The present study investigates the decision making towards CABG or PCI in patients suffering from left main stenosis in clinical practice by analyzing a complete national dataset. We compare in-hospital death and further complications such as bleeding, stroke, postoperative delirium, and prolonged ventilation between both treatment options. Furthermore, the resource utilization is determined.

## Methods

Since 2005, data on all hospitalizations in Germany have been available for scientific use via the Diagnosis Related Groups statistics collected by the Research Data Center of the Federal Bureau of Statistics (DESTATIS). These hospitalization data, including diagnoses and procedures, are a valuable source of representative nationwide data on the in-hospital treatment of patients. This database represents a virtually complete collection of all hospitalizations in German hospitals that are reimbursed according to the Diagnosis Related Groups system. From this database, we extracted data on patients with left main stenosis (I25.14) who underwent either isolated CABG or coronary stent implantation in 2018, the most recent available year for analysis. In order to identify comparable groups, patients with acute coronary syndromes such as in-stent stenosis, NSTEMI, STEMI or unstable angina pectoris were excluded from the dataset. Emergency admission are coded whenever patients are admitted non-elective via ambulance or emergency department. Furthermore, patients with any other kind of concomitant heart valve surgery during the same episode of hospitalization were also excluded. A complete list of procedure codes as well as a more detailed discussion of the validity of the data source may be found in Table S1.

Our study did not involve direct access by the investigators to data on individual patients but only access to summary results provided by the Research Data Center. Therefore, approval by an ethics committee and informed consent were determined not to be required, in accordance with German law.

All summary results were anonymized by DESTATIS. In practice, this means that any information allowing the drawing of conclusions about a single patient or a specific hospital was censored by DESTATIS to guarantee data protection. Moreover, in order to prevent the possibility to draw conclusions to a single hospital, the data are verified and situationally censored by DESTATIS in those cases.

### Endpoints

The analysis focused on seven different endpoints: in-hospital mortality, bleeding events, stroke, postoperative delirium, mechanical ventilation exceeding 48 h, length of hospital stay and reimbursement. Stroke and postoperative delirium were defined using ICD, tenth revision (ICD-10) codes (secondary diagnosis I63* or I64 and F05*, respectively). Bleeding was defined as requiring a transfusion of > 5 units of red blood cells and identified using OPS-codes (8–800.c1 to 8–800.cr). In-hospital mortality, length of mechanical ventilation, and length of hospital stay were part of DESTATIS’ main set of variables. For all other comorbidities, the existing anamnestic or acute distinctive codes were used (we have discussed OPS and ICD codes in detail previously^8^).

For calculation of the estimated logistic EuroSCORE (European System for Cardiac Operative Risk Evaluation), we were able to populate all fields except for critical preoperative state and left ventricular function. In these, we assumed an inconspicuous state (i.e., no critical preoperative state and no left ventricular dysfunction) and thus calculated a best-case scenario.

### Statistical analysis

In previous studies [20, 22], we identified 20 baseline characteristics to describe risk profiles between procedural groups. Patients were treated in clinical practice and not randomized to the two treatment options (CABG or coronary stent implantation). Therefore a logistic or linear regression model were used including 20 baseline patient characteristics as potential confounders (Table 1). Nonelective emergency admission of the procedure was also added as confounder. To account for the correlation of error terms of patients treated in the same hospital, a random intercept was included at the center level. See Table S2 for results of the different regression analyses.

**Table 1:** Baseline characteristics 2018

	CABG	Stent	*p* value
*N*	1318	8920	
Logistic EuroSCORE^1^, mean/SD	4.85 ± 4.65	8.77 ± 8.45	< 0.001
Age in years, mean/SD	68.63 ± 9.40	72.58 ± 9.87	< 0.001
Female %	16.24%	22.05%	< 0.001
NYHA II, %	< 0.30%*	11.63%	< 0.001
NYHA III or IV, %	23.60%	16.69%	< 0.001
Hypertension, %	74.81%	63.91%	< 0.001
Previous MI within 4 months, %	2.50%	5.78%	< 0.001
Previous MI within 1 year, %	1.59%	2.57%	0.032
Previous MI after 1 year, %	< 0.30%*	10.01%	< 0.001
Previous CABG, %	< 0.30%*	16.14%	< 0.001
Previous cardiac surgery, %	< 0.30%*	18.06%	< 0.001
Peripheral vascular disease, %	10.24%	8.17%	< 0.001
Carotid disease, %	< 0.30%*	3.11%	< 0.001
COPD, %	< 0.30%*	7.59%	< 0.001
Pulmonary hypertension	< 0.30%*	5.62%	< 0.001
Renal disease, GFR < 15%, %	< 0.30%*	1.74%	< 0.001
Renal disease, GFR < 30%, %	< 0.30%*	2.22%	< 0.001
Atrial fibrillation, %	33.16%	21.61%	< 0.001
Diabetes, %	32.32%	32.43%	0.936
Emergency Admission	< 0.30%*	25.81%	< 0.001

*SD* standard deviation, *NYHA* New York Heart Association class, *MI* myocardial infarction, *CABG* coronary artery bypass grafting, *COPD* chronic obstructive pulmonary disease, *GFR* glomerular filtration rate

*Groups with < 3 cases are blanked out by the Research Data Centers of the Federal Bureau of Statistics for reasons of anonymity. Thus, the share of those patients is under 0.3%^1^ For calculation of the estimated logistic EuroSCORE (European System for Cardiac Operative Risk Evaluation), we were able to populate all fields except for critical preoperative state and left ventricular function. In these, we assumed an inconspicuous state (i.e., no critical preoperative state and no left ventricular dysfunction) and thus calculated a best-case scenario.

No imputation for missing values could be conducted due to the absence of codes indicating that data were missing. If the patient’s electronic health record did not include information on a clinical characteristic, it was assumed that that characteristic was not present. Furthermore, no adjustment for multiple testing was carried out. Thus, *p* values may not be interpreted as confirmatory but are descriptive in nature and inferences drawn from the 95% confidence intervals may not be reproducible.

All analyses were performed with Stata 16 (StataCorp, College Station, Texas, USA).

## Results

### Treatment decision in clinical practice in 2018

**Fig. 1:**
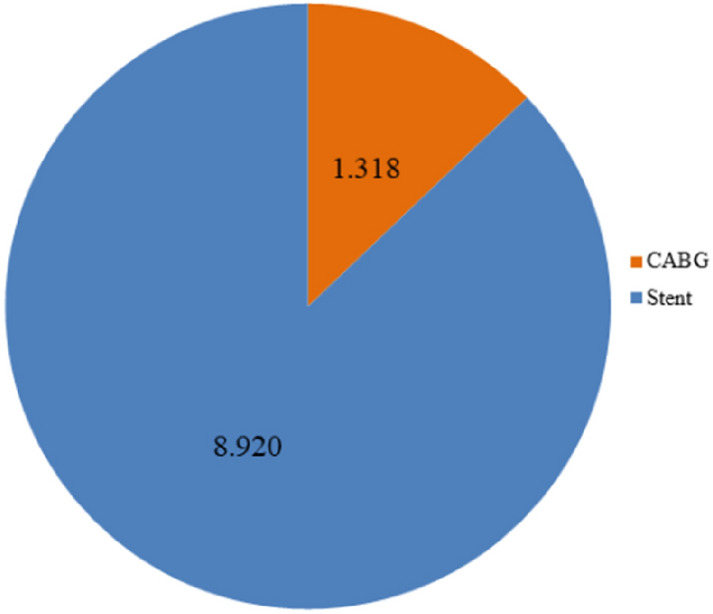
CABG vs. stent distribution in clinical practice in 2018. All cases with diagnosis of left main stenosis without STEMI, NSTEMI, unstable angina, in-stent stenosis, and without any other concomitant heart valve surgery were identified

All 10,238 cases with diagnosis of left main stenosis receiving either CABG or stent in Germany 2018 were identified. Patients with STEMI, NSTEMI, unstable angina, in-stent stenosis, and any kind of concomitant heart valve surgery were excluded. 87% of patients with chronic coronary syndrome and left main coronary artery disease were assigned to stent implantation (Fig. 1).

### Baseline characteristics

The stent group was older, had more relevant comorbidities such as coronary, carotid, or peripheral artery disease, and renal or pulmonary disease. Moreover, cases assigned to stent were more often admitted as emergency cases, but CABG patients were more frequently in NYHA class III or IV. The share of female patients was 16% in the CABG group and 22% in the stent group. In summary, the stent group was at higher operative risk as assessed by the logistic EuroSCORE (4.85 ± 4.65 vs. 8.77 ± 8.45, *p* < 0.001, Table 1).

### Unadjusted in-hospital outcomes

The in-hospital mortality of patients after CABG was 1.7% and after stent 2.4% in patients with left main disease (*p* = 0.109). Relevant bleedings occurred in 4.8% of patients after CABG and 2.4% of patients after stent (*p* < 0.001). The rate of stroke was 0.83% in the CABG group and 0.45% in the stent group (p < 0.001). Naturally, postoperative delirium (9.0% vs. 1.9%, *p* < 0.001) and prolonged mechanical ventilation (4.7% vs. 2.1%, *p* < 0.001) were more frequently observed in the CABG group (Table 2).

**Table 2:** Unadjusted in-hospital outcomes 2018

	CABG	Stent	*p* value
*N*	1318	8920	0.109
In-hospital mortality	1.67%	2.38%	< 0.001
Bleeding	4.78%	1.17%	0.063
Stroke	0.83%	0.45%	< 0.001
Postoperative delirium	9.03%	1.91%	< 0.001
Mechanical ventilation > 48 h	4.70%	2.09%	< 0.001

### Multivariable analysis of in-hospital outcomes

**Fig. 2:**
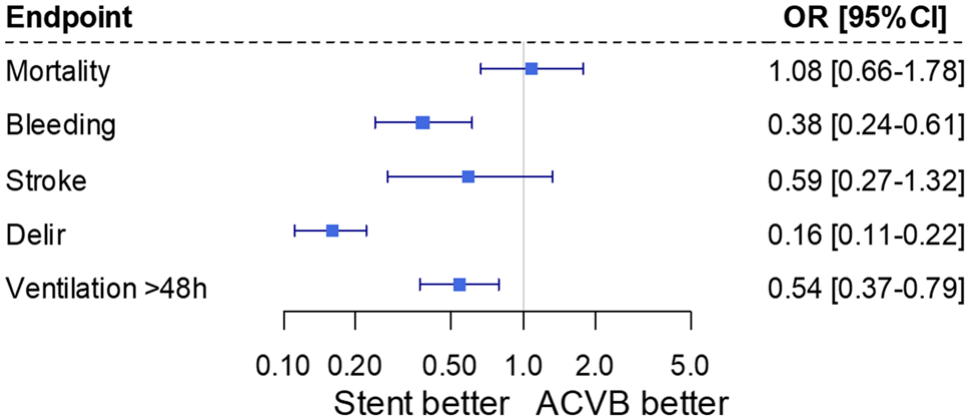
Risk-adjusted in-hospital outcomes. Results of multivariable logistic regression analyses with predefined baseline patient characteristics included as potential confounders (all covariates listed in Table 1)

After adjusting for all baseline characteristics, risk for in-hospital mortality (OR 1.08, [95% CI 0.66; 1.78], *p* = 0.748) and stroke (OR 0.56 [0.27; 1.32], *p* = 0.199) did not differ between CABG and stent in patients suffering from left main disease and chronic coronary syndrome. According to the results of the unadjusted analysis, the risk of other complications was higher in the CABG group. Stent implantation was associated with significantly reduced risk for relevant bleeding (OR 0.38 [0.24; 0.61], *p* < 0.001), prolonged ventilation time (OR 0.54 [0.37; 0.79], *p* = 0.002), and postoperative delirium (OR 0.16 [0.11; 0.22], *p* < 0.001, Fig. 2).

### Resource utilization CABG vs. stent

Naturally the invasiveness is usually higher in CABG than in left main stenting. In order to elucidate the resource utilization of both treatment options we determined length of hospital stay and reimbursement. Patients undergoing CABG were hospitalized 7.3 days longer (CABG 13.04 ± 9.26, stent 5.71 ± 7.60 days, *p* < 0.001). Accordingly, the costs of CABG were three times higher than those of left main stenting (CABG: 17,573.78 ± 11,153.57€; stent: 5892.94 ± 8695.00€, *p* < 0.001). Even after adjusting for risk factors, PCI was associated with shorter hospital stay (− 6.78 days [− 5.86; − 7.71], *p* < 0.001) and lower costs (− 10,035 € [− 11,500; − 8570], *p* < 0.001). Thus, the resource utilization is lower in patients with left main stenosis undergoing left main stenting (Table 3).

**Table 3:** Ressource utilization

	Unadjusted		Adjusted
	CABG	Stent	Stent instead of CABG Coefficient [95% CI]
Length of hospital stay in days	13.04 ± 9.26	5.71 ± 7.60	− 6.78 [− 5.86 to − 7.71] *p* < 0.001
Reimbursement in Euro	17,573.78 ± 11,153.57	5892.94 ± 8695.00	− 10,035 [− 11,500 to − 8570] *p* < 0.001

## Discussion

The present study including 10,238 cases shows that in-hospital mortality was not significantly different between PCI and CABG in patients with left main coronary artery disease in clinical practice. It also shows that the vast majority of patients with left main stenosis underwent stenting. Patients assigned for left main stenting had lower risk for in-hospital complications such as bleeding, prolonged mechanical ventilation, and postoperative delirium. PCI utilized less health-economic resources.

The ESC guidelines rank left main stenting as equal to CABG for patients with stable coronary artery disease and low SYNTAX score, with an IA recommendation for both [16]. The American revascularization guidelines rate left main PCI as an appropriate alternative to CABG in patients with low-to-intermediate anatomical complexity [19]. The present analysis shows that left main stenting is the main treatment approach in current clinical practice in Germany. In 2018 almost 90% of patients with chronic coronary syndrome were assigned for left main stenting, indicating the importance of PCI in the care of patients suffering from left main stenosis in clinical practice.

The decision making for PCI vs. CABG should be made by an interdisciplinary approach within the Heart Team to provide an individualized treatment concept. Anatomical and technical aspects, patient’s preferences, and clinical characteristics such as age and comorbidities should be considered [16]. In the present cohort, the patients assigned for left main stenting were older, had more comorbidities, and consequently a higher logistic EuroSCORE. This might account for the high proportion of stenting in clinical practice.

Randomized clinical trials provide evidence for clinical guidelines. However, patients are carefully selected before participation. Thus, marked differences occur between clinical trials and daily practice. Therefore the present data-set is a reliable tool to assess guideline adherence and outcomes in clinical practice, although long-term follow-up data is not available [20, 22]. Without adjustment we found slightly increased in-hospital mortality after PCI. With respect to the increased operative risk in patients undergoing PCI, we performed a multivariate logistic regression to allow a comparison of both treatment strategies and found that after risk adjustment the risk for death did not differ. This is in line with data from the NOBLE, EXCEL, PRECOMBAT, and SYNTAX trials, where short-term and long-term mortality was similar in patients suitable for both CABG and left main stenting [2, 14, 17, 26]. In a meta-analysis summarizing these trials, mortality did not differ even after long-term follow-up of up to 10 years [2]. The present analysis confirms that the results of randomized trial are translated into the clinical practice: in-hospital mortality of PCI and CABG in patients with CCS and left main stenosis are comparable. Accordingly, risk for stroke also did not significantly differ in randomized trials and in the present analysis. However, we could analyze outcomes which are not reflected in the randomized controlled trials. Due to the more invasive nature of CABG it is not surprising that the risk for relevant bleeding, prolonged ventilation, and postoperative delirium was higher in the CABG group. Those factors increase the utilization of hospital resources such as stay on intensive care unit. In particular during the COVID pandemic this may be an important benefit of left main stenting compared to CABG [3, 4]. Although those complications did not result in increased in-hospital mortality, they may be associated with long-term sequelae: blood transfusion can cause transfusion reactions [25], mechanical ventilation can result in ventilator-associated pneumonia [13], and postoperative delirium may induce or accelerate long-term cognitive decline [11]. The risk for repeated revascularization was consistently increased for patients undergoing left main stenting in all clinical trials during the follow-up period. Due to the nature of the present data set, we analyzed only in-hospital outcomes. Thus, we cannot report whether the observed reduction of in-hospital complications in patients undergoing left main stenting outweigh repeated revascularization during follow-up as seen in randomized controlled trials. Nevertheless, integrating results of randomized controlled trial with the present analysis shows that left main stenting is safe with even some advantages in respect to in-hospital complications in clinical practice beyond clinical trials. Convincing results in clinical practice and positive results clinical trials might explain why left main stenting markedly overtook CABG compared to guidelines recommendations.

Considering the comparable outcomes with respect to mortality and other complications, we analyzed the resource utilization of CABG vs. stent in patients with left main stenosis. Unsurprisingly given the less invasive approach of stenting and lower rate of in-hospital complications, the length of hospital stay was shorter by 7.3 days. Costs per case were over 11,000 Euro lower in the PCI group. This is in line with analyses of the SYNTAX study where left main stent was associated with more favorable costs in particular in low-risk patients [6]. The economic aspects become more important advanced treatment strategies which are associated with increased costs.

### Limitations

In our study, we analyze a special subset of patients: those with CCS and a left main stenosis, but without concomitant indications for surgery of valvular disease. Therefore, we cannot transfer our findings to patients with acute coronary syndrome or three-vessel disease, or to patients with relevant other structural heart diseases.

The comparison of CABG versus stents in left main stenosis assumes that both approaches are possible. The present data source does not provide anatomical or echocardiographic data; therefore the SYNTAX score cannot be calculated. The calculated logistic EuroSCORE is an approximation since a conservative or ‘best-case scenario’ estimate is applied. The administrative data are designed to report diagnoses and procedures in order to trigger reimbursement. The competing interests of hospitals and health insurers ensure a high level of data reliability and quality, but coding errors cannot be completely ruled out.

In addition, long-term follow-up data are missing, as DESTATIS provides no longitudinal data or cross-links with other clinical or administrative datasets. This is of special interest with regard to repeated revascularizations, which can be more often found in patients treated with stents compared to CABG, as discussed above. Finally, this analysis relies on data from the German healthcare system and other countries’ experiences may differ.

## Conclusion

The present analysis shows that in CCS patients left main stenting appears the mostly used standard of care in clinical practice in Germany, with favorable in-hospital outcomes. In-hospital mortality and stroke rates are similar between the two procedures, but bleeding, postoperative delirium, and prolonged ventilation were lower in patients undergoing left main stenting compared to CABG. However, we cannot conclude on mid- and long-term outcomes of repeated revascularization in patients with PCI or CABG.

In summary, concerning in-hospital outcomes, left main stenting is a safe and essential treatment option for patients suffering from left main coronary artery disease at reasonable economic cost.

## Data Availability

The dataset is available at the German Bureau of statistics.
